# Editorial: Neurotropism of Parasites and the Immune Responses

**DOI:** 10.3389/fimmu.2021.775666

**Published:** 2021-10-01

**Authors:** Miriam Postan, Martin E. Rottenberg

**Affiliations:** ^1^ Consejo Nacional de Investigaciones Científicas y Tecnológicas (CONICET), Buenos Aires, Argentina; ^2^ Instituto Nacional de Parasitología "Dr. Mario Fatala Chabén", Administración Nacional de Laboratorios e Institutos de Salud "Dr. Carlos G. Malbran" (ANLIS)/Malbran, Buenos Aires, Argentina; ^3^ Department of Microbiology, Tumor and Cell Biology, Karolinska Institutet, Stockholm, Sweden

**Keywords:** parasites, neurotropism, blood brain barrier, immune responses, fungi

Parasitic infections of the central and peripheral nervous systems represent a significant source of morbidity and mortality, especially in humans living in low-to-middle income endemic countries. Examples of these are cerebral malaria, neurocysticercosis, African and American trypanosomiasis and toxoplasmic encephalitis, which may have a predilection for the CNS.

In spite of the known association between parasitic infections and specific neurological, cognitive and mental disorders, the exact mechanisms by which “neurotropic” parasites (i) enter the central nervous system (CNS) *via* the blood-brain barrier (BBB) and *via* the blood-CSF barrier and (ii) induce neurological damage have not been fully elucidated yet.

The study of parasitic brain infections is intertwined with that of neuroinflammation and brain damage. A primary role of neuroinflammation is to protect the CNS from insults, including invasion and attack by infectious agents. Pathogens can benefit from immune responses to promote neuroinvasion, for example, when neuroinflammation facilitates the opening of physical barriers, or when the pathogen develops Trojan horse strategies by using activated cells to enter the brain. Once inside the brain, pathogens are difficult to dislodge and cause CNS dysfunctions. This is the case for various protozoan parasites including the intracellular *Toxoplasma* and the extracellular African trypanosomes causing chronic CNS infections. Some of these neuroinvasion mechanisms were also described for fungi such as *Cryptococcus neoformans, Aspergillus fumigatus* and *Candida albicans* (1).

*Toxoplasma* infects one third of the human population globally. In this Research Topic (RT), Schlüter and Barragan discuss how immune cells can help the spread of *Toxoplasma* and are programmed by infection of *Toxoplasma* to spread into the CNS, and how parasite specific Tcell-mediated immune responses control but do not eradicate the infection. Additionally, the function of brain resident cell populations is delineated, i.e., how neurons, astrocytes and microglia serve both as target cells for the parasite but also actively contribute to the immune response is discussed. Mounting evidences that chronic CNS toxoplasmosis may impact in neuropsychiatric diseases or modulate cognitive functions are also presented. Focusing on ocular toxoplasmosis, S. Lie et al. analysed transcriptomes from primary human retinal pigment epithelial cells and cell lines infected with *T. gondii* strains *in vitro*. *T. gondii* infection triggered the immunological activation of *T. gondii*-infected retinal pigment epithelial cells, cells that form the blood - ocular barrier.

Human African trypanosomiasis (HAT) or sleeping sickness has been recognized as a scourge in sub-Saharan Africa for centuries. The disease, caused by the protozoan parasite *Trypanosoma brucei*, is a major cause of mortality and morbidity in animals and man. Infection with this parasite results in an early hemolymphatic-stage followed by a late encephalitic-stage when the parasites migrate into the CNS. Herein, Kennedy and Rodgers present an overview of the clinical features of HAT, with special emphasis on specific features of the late stage of the disease. The epidemiological implications of previously unrecognized asymptomatic individuals is discussed as well as biological features of *T. brucei*, new drug candidates and diagnostic tools.

Circumventricular organs (CVOs), neural structures located around the third and fourth ventricles, harbor, similarly to the choroid plexus, vessels devoid of a continuous nonfenestrated barrier, which enable them to sense immune-stimulatory molecules in the blood circulation, but may also increase chances of exposure to microbes. However, attacks to CVOs by microbes are rarely described. M. Bentivoglio et al. highlight that *T. brucei* circulating in the bloodstream can attack the CVOs and choroid plexus, providing a route for brain penetration. From the choroid plexus trypanosomes can seed into the ventricles and initiate accelerated infiltration of T cells and parasites in periventricular areas. Trypanosomes can also invade CVOs such as the median eminence located in the base of the third ventricle, and by crossing the border into the BBB-protected hypothalamic arcuate nuclei. Activity in arcuate nucleus neurons affects sleep/wake behavior. Data showing that trypanosome invasion of the CVOs can contribute to triggering initial and distinct CNS imbalances of HAT and the mechanisms involved are discussed.

Other pathogens, such as *Plasmodium*, cause severe alterations and inflammation in the brain vasculature, without entering the brain parenchyma. Sequestration of *Plasmodium*-infected red blood cells in brain microvessels is a hallmark of cerebral malaria pathology. The role of innate immune responses in cerebral malaria is addressed here by Pais and Penha-Goncalves. They describe how recognition of various parasite-derived molecules by brain endothelial cells innate immune receptors mediates their activation, and trigger inflammatory responses that lead to microcirculatory and coagulation disturbances and to altered vascular permeability impairing BBB integrity.

Metazoan parasites have also evolved mechanisms for invading cerebrospinal tissues. They may invade the CNS *via* hematogenous spread of larval stages to small vessels, by *in situ* deposition or embolism of eggs following anomalous migration of adult worms to the CNS, attaching to the nasal neuroepithelium and penetration *via* the olfactory nerve pathway, or directly invading the neural skull and intervertebral foramina. Neurocysticercosis, caused by blood dissemination of *Tenia solium* larval stages (cysticerci), is the most common helminth infection of the CNS in humans. Clinical presentation and the immunology of neurocysticercosis vary according to the type and location of cysts inside the brain parenchyma or in the cerebral ventricles and subarachnoid spaces (extraparenchymal) (2). In this RT, Toledo et al discuss the need of finding sensitive and specific biomarkers that could predict the intensity of inflammatory response to drug treatment.

Although fungal infections of the CNS are uncommon, prolonged systemic circulation of fungi can affect the CNS, thus contributing to morbidity and mortality of the infected patients (3). This RT includes an original research article by M. D’Alessandre Sanches et al., who show the ability of 3 *non-albicans Candida* species to spread to the CNS in the mouse model, as these species are becoming more frequently involved in CNS candidiasis infections. They show that *Candida glabrata*, *Candida krusei* and *Candida parapsilosis* are able to disseminate to the CNS and promote local inflammation in both immunocompetent and immunosuppressed mice early after infection.

Parasitic infections also affect the peripheral nervous system (PNS), probably *via* the production of parasitic molecules (such as cysteine proteases from Entamoeba) and/or *via* the immune responses stimulated by the infection. The altered activity of peripheral neurons in these infections, through gut-brain communication may modulate immune activity. Distress in the enteric nervous system plays a key role in the pathophysiology of diarrhea upon exposure to several enteric protozoan and metazoan parasitic infections (i.e. *Giardia. sp*; *E. histolytica*). This distress has also been indicated to play a role in echagasic megasyndromes and post-infectious complications.

The roles of systemic innate and adaptive immune responses in the perpetuation or eradication of parasitic infections in the brain is interconnected with the pathogenesis of parasitosis. Understanding these roles will help advance the development of preventive measures against parasite-induced neurological diseases. This RT has covered recent advances in the pathogenesis of parasites affecting the CNS and the PNS as well as the role of the immune responses in these infections, highlighting prospects for potential interventions.

## Author Contributions

All authors listed have made a substantial, direct, and intellectual contribution to the work and approved it for publication.

## Funding

Supported by the Swedish Foundation for International Cooperation in Research and Higher Education.

## Conflict of Interest

The authors declare that the research was conducted in the absence of any commercial or financial relationships that could be construed as a potential conflict of interest.

## Publisher’s Note

All claims expressed in this article are solely those of the authors and do not necessarily represent those of their affiliated organizations, or those of the publisher, the editors and the reviewers. Any product that may be evaluated in this article, or claim that may be made by its manufacturer, is not guaranteed or endorsed by the publisher.

## References

[1] Strickland AB Shi M. Mechanisms of Fungal Dissemination. Cell Mol Life Sci (2021) 78(7):3219–38. doi: 10.1007/s00018-020-03736-z PMC8044058 33449153

[2] Prodjinotho UF Lema J Lacorcia M Schmidt V Vejzagic N Sikasunge C. Host Immune Responses During Taenia Solium Neurocysticercosis Infection and Treatment. PLoS Negl Trop Dis (2020) 14(4):e0008005. doi: 10.1371/journal.pntd.0008005 32298263 PMC7162612

[3] Cesaro S Tridello G Castagnola E Calore E Carraro F Mariotti I. Retrospective Study on the Incidence and Outcome of Proven and Probable Invasive Fungal Infections in High-Risk Pediatric Onco-Hematological Patients. Eur J Haematol (2017) 99(3):240–8. doi: 10.1111/ejh.12910 28556426

